# ALDH2 mitigates LPS-induced cardiac dysfunction, inflammation, and apoptosis through the cGAS/STING pathway

**DOI:** 10.1186/s10020-023-00769-5

**Published:** 2023-12-20

**Authors:** Haoran Liu, Qin Hu, Ke Ren, Pengxin Wu, Yang Wang, Chuanzhu Lv

**Affiliations:** 1 Emergency and Trauma College, Hainan Medical University, Haikou, China; 2 Key Laboratory of Emergency and Trauma of Ministry of Education, Hainan Medical University, Haikou, China; 3 Research Unit of Island Emergency Medicine, Chinese Academy of Medical Sciences (No. 2019RU013), Hainan Medical University, Haikou, China; 4 Emergency Medicine Center, Sichuan Provincial People’s Hospital, University of Electronic Science and Technology of China, Chengdu, China

**Keywords:** ALDH2, cGAS/STING, Lipopolysaccharide, Cardiac dysfunction, Inflammation, Apoptosis

## Abstract

**Background:**

Sepsis is a severe syndrome of organ dysfunction that often leads to cardiac dysfunction and endangers life. The role of mitochondrial aldehyde dehydrogenase 2 (ALDH2) in LPS-induced myocardial injury is unclear. The purpose of this study was to assess the role of ALDH2 in lipopolysaccharide (LPS)-induced myocardial injury and the regulatory mechanism and to identify potential therapeutic strategies for treating this condition.

**Methods:**

An in vivo model was established by 12 h of LPS (10 mg/kg, intraperitoneal injection) stimulation, and an in vitro model was generated by stimulating H9C2 cells with LPS (10 μg/ml) for 12 h. We then used the ALDH2 activator Alda-1 and the ALDH2 inhibitor daidzin to assess their effects on LPS-induced cardiac injury. Cardiac function in mice was evaluated by using cardiac ultrasound. We used various methods to evaluate inflammation, apoptosis, and oxidative stress, including ELISA, flow cytometry, JC-1 staining, Western blotting, and DCFH-DA staining. Additionally, we used a small interfering RNA (siRNA) to knock down cyclic GMP-AMP synthase (cGAS) to further investigate the relationship between ALDH2 and cGAS in LPS-induced cardiac injury.

**Results:**

LPS-induced cardiac dysfunction and increased the levels of the cardiac injury markers creatine kinase-MB (CKMB) and lactate dehydrogenase (LDH) in vivo. This change was accompanied by an increase in reactive oxygen species (ROS) levels, which exacerbated the oxidative stress response and regulated apoptosis through cleaved caspase-3, BAX, BCL-2. The expression of inflammatory cytokines such as IL-6/IL-1β/TNF-α was also upregulated. However, these effects were reversed by pretreatment with Alda-1 via the inhibition of cGAS/stimulator of interferon genes (STING) signaling pathway. Interestingly, LPS, Alda-1 and daidzin altered the activity of ALDH2 but did not regulate its protein expression. Knocking down cGAS in H9C2 cardiomyocytes alleviated LPS-induced cardiac inflammation, apoptosis, and ROS production and weakened the synergistic effect of daidzin.

**Conclusion:**

We demonstrated that ALDH2 alleviated LPS-induced cardiac dysfunction, inflammation, and apoptosis through the cGAS/STING signaling pathway, thereby protecting against LPS-induced cardiac injury. This study identifies a novel therapeutic approach for treating sepsis-induced cardiomyopathy (SIC).

**Supplementary Information:**

The online version contains supplementary material available at 10.1186/s10020-023-00769-5.

## Introduction

Sepsis is a serious threat to human health and safety due to its complex pathogenesis, high mortality and complications (Beesley et al. 2018; Mervyn et al. 2016). Clinically, reversible cardiac dysfunction is observed in approximately 40% to 60% of sepsis patients (Lu et al. 2020). Considering that clinical trials targeting specific proinflammatory cytokines with therapeutic antibodies have largely ended in failure and the absence of standardized diagnostic and therapeutic criteria, the incidence and mortality rates of sepsis-induced cardiomyopathy (SIC) remain high (Mervyn et al. 2016; Lu et al. 2020; Ehrman et al. 2018; Ouyang et al. 2020; Boyd et al. 2006; V, Kumar. 2020). Conversely, increasing evidence suggests a pathological association between SIC and mitochondria dysfunction (Ji et al. 2021; Zheng et al. 2017; Durand et al. 2017). Myocardial cells in SIC exhibit structural changes in their mitochondria (Takasu et al. 2013), led an increase in mitochondrial membrane permeability, further increased reactive oxygen species (ROS) level and activated mitochondrial apoptosis pathway (Tan et al. 2019; Haileselassie et al. 2017). Therefore, how to effectively maintain mitochondrial function and protect myocardial cells involved in triggering cardiac dysfunction during SIC, promote myocardial functional recovery, and reduce myocardial apoptosis, and inflammatory response, remains a key focus and challenge in current research (Martin et al. 2016; Galley 2011; Liu et al. 2023).

Aldehyde dehydrogenase 2 (ALDH2), an enzyme primarily located in the mitochondria, plays a crucial role in the detoxification of acetaldehyde and endogenous lipid aldehydes. Research has shown that ALDH2 protects against cardiovascular diseases (CVDs) (Chen et al. 2014), and its activity is significantly negatively correlated with the myocardial infarction area (Zhong et al. 2019). Studies have shown that increasing ALDH2 activity can significantly improve myocardial function (Pang et al. 2019; Wang et al. 2011). The overexpression of ALDH2 has been shown to ameliorate chronic alcohol-induced myocardial hypertrophy, contractile dysfunction (Ge et al. 2011; Li et al. 2009) and obesity-induced cardiomyopathy (Wang et al. 2018) by reducing mitochondrial damage. Recent study also indicated that activation of ALDH2 can inhibit oxidative stress and inflammation, preventing myocardial fibrosis and cell apoptosis induced by a high glucose environment (Kang et al. 2020). However, the specific role of ALDH2 in sepsis-induced myocardial injury remains unclear.

Cyclic GMP-AMP synthase (cGAS) is a widely distributed signaling-associated pattern recognition receptor (PRR) located in the cytoplasm (Tan et al. 2021). cGAS can recognize double-stranded DNA (dsDNA) derived from pathogens, the cell nucleus, or mitochondria, and is associated with various inflammatory diseases (Zhao et al. 2020). When cells or tissues are stimulated with lipopolysaccharide (LPS), abnormal release of dsDNA can serve as a damage-associated molecular pattern (DAMP) to activate cGAS (Li et al. 2022). However, excessive activation of cGAS can lead to downstream reactions involving the stimulator of interferon genes (STING), promoting cellular metabolism, apoptosis, autophagy, and contributing to the occurrence and progression of inflammatory diseases such as acute pancreatitis and liver injury (Gui et al. 2019; Cai et al. 2020). In a recent study, it was found that melatonin has a protective effect against cardiac dysfunction and mitochondrial damage induced by APP/PS1 mutation. However, this protective effect is attenuated cGAS/STING is knocked down using si-RNA or ALDH2 is knocked out (Wang et al. 2020). The potential mechanisms of cGAS/STING and ALDH2 in LPS-induced systemic inflammatory response syndrome are unclear and warrant further investigation.

In this study, we found that increasing ALDH2 activity can inhibit the expression of the cGAS/STING signaling pathway, reduce ROS production, alleviate inflammation and apoptosis, and improve cardiac dysfunction. Furthermore, we also observed that knocking down cGAS attenuated the effects of ALDH2 and LPS, indicating that ALDH2 exerts its protective role in LPS-induced septic cardiomyopathy through the cGAS/STING signaling pathway. Our study provides groundbreaking insights and robust data support for the diagnosis and treatment of SIC.

## Materials and methods

### Reagents and antibodies

The details of the drugs and related reagents used in this study are presented in Additional file 1: Table S1.

### Animals

A total of 48 male C57BL/6 mice were purchased from Byrness Weil Biotech Ltd. (License number: SCXK (Xiang) 2019-0004). The mice were of SPF-grade and had body weight of approximately 20 g. The mice were housed under SPF-grade conditions throughout the study. The mice were provided free access to food and water and were kept in a controlled environment (temperature: 24 ± 2 °C, humidity: 55 ± 5%, light/dark cycle: 12 h/12 h). The mice were acclimatized to the housing conditions for one week before the experiment. The welfare of the animals was strictly maintained according to the international regulations on experimental animals. The animal experiments were conducted according to protocols approved by the ethics committee at Number 326, 2023 of Sichuan Province People’s Hospital.

### In vivo model

Forty-eight mice were randomly divided into four groups (12 mice in each group): the control group, lipopolysaccharide (LPS) group, control + Alda-1 (Alda-1 is an ALDH2 activator) group, and Alda-1 + LPS groups. The mice were subjected to a 12 h fasting period with free access to water prior to the start of the experiment. In the control group, 0.1 ml of physiological saline was intraperitoneally injected. In the LPS group, the mice were intraperitoneally injected with 10 mg/kg *Escherichia coli* 055: B5 LPS for a treatment duration of 12 h (Li et al. 2019, 2020). In the Alda-1 group, the mice were intraperitoneally injected with 10 mg/kg Alda-1 for a treatment duration of 12 h (Hu et al. 2015; Ji et al. 2016). In the Alda-1 + LPS group, mice were first intraperitoneally injected with Alda-1 (10 mg/kg) for 12 h, followed by LPS treatment (Ji et al. 2016).

### Echocardiographic assessment

To facilitate examination, we removed the fur from the chest and then allowed the mice to inhale isoflurane. The mice were placed on a heated pad to maintain a body temperature of 37 °C. Cardiac examination was performed using a portable color Doppler ultrasound system (M90 SCI) with an ultrasound probe (L20-5 s; Mindray Medical International Ltd, China). Two-dimensional guided M-mode measurements of the left ventricular (LV) diameter were acquired from short-axis views at the level of the myocardium. Left ventricular ejection fraction (LVEF) and fractional shortening (FS) were calculated utilizing computer algorithms. Measurement of Left Ventricular Dimensions: Measure the left ventricular end-diastolic diameter (LVEDD) and left ventricular end-systolic diameter (LVESD) from the segmented wall boundaries. Calculation of LVEF: Calculate the left ventricular end-diastolic volume (LVEDV) and left ventricular end-systolic volume (LVESV) using the measured dimensions. Then, compute the LVEF using the formula: LVEF = ((LVEDV − LVESV) / LVEDV) × 100%. Calculation of FS: Calculate the fractional shortening (FS) using the formula FS = ((LVEDD − LVESD)/LVEDD) × 100%. All echocardiograms were performed and interpreted by trained technicians, and the data obtained were the average of at least three cardiac cycles. The researchers performing and interpreting the echocardiograms were completely blinded to the specific groupings of the mice.

### Cellular model

The H9C2 cell line derived from rat cardiomyocytes [H9C2( 2–1) (CL-0089)] was purchased from Procell Life Science & Technology Co., Ltd. (Wuhan, China).

### In vitro model

We used H9C2 cells within ten passages for cell culture. The medium was changed when the cells reached approximately 60% confluence. The cell models were divided into three plates: Alda-1 (CON, LPS, Alda-1, Alda-1 + LPS), daidzin (CON, LPS, daidzin (daidzin is an ALDH2 inhibitor), daidzin + LPS), cGAS-siRNA (CON, LPS, cGAS-siRNA + LPS, cGAS-siRNA + daidzin + LPS). The LPS treatment duration was 12 h, Alda-1 and daidzin pretreatment was performed for 2 h, and siRNA transfection was carried out for 48 h. The final concentrations of LPS, Alda-1 and daidzin were 10 μg/ml (Wang et al. 2019), 20 μM (Ji et al. 2016) and 48 μM (Ji et al. 2016), respectively.

### siRNA transfection

Cell culture was performed in a 6-well plate per the manufacturer’s instructions. When the cells reached 30–40% confluence, the medium was changed to Opti-MEM™ I with reduced serum, and Lipofectamine 3000 was to transfect NC-small interfering RNA and cGAS-siRNA. The medium was replaced with complete culture medium after 6 h.

### Measurement of ALDH2 activity

All procedures were performed in strict accordance with the manufacturer’s instructions. The principle behind this is that ALDH2 catalyzes the reaction between acetaldehyde and NAD^+^, and the change in absorbance of NADH at 340 nm can be used to calculate the activity of ALDH2.

### CCK8 assay

All procedures were performed in strict accordance with the manufacturer's instructions.

### Histopathological examination

After obtaining blood from the mouse heart, the heart tissue was quickly removed, and the apex of the heart was placed in tissue fixative for Hematoxylin–eosin staining (HE)/ Immunohistochemistry (IHC) staining. The remaining parts of the heart were rinsed in saline and then frozen in liquid nitrogen before being stored at − 80 °C for protein expression analysis.

### TUNEL staining

Apoptosis was evaluated using the ApopTag^®^ In Situ Apoptosis Detection Kit, which employs terminal deoxynucleotidyl transferase (TdT)-mediated dUTP nick-end labeling (TUNEL) staining. Following staining, the tissue sections were examined under a fluorescence microscope (Olympus Corporation) to visualize and quantify apoptotic cells. This method allows for the accurate detection and assessment of apoptosis in the tissue samples (analyzed using Image-Pro Plus 6.0).

### CK-MB and LDH detection

The extracted blood was placed in a centrifuge tube and allowed to stand at 4 ℃ for 30 min. The blood was then centrifuged at 3000 × g for 15 min, and the supernatant was collected and stored at − 80 °C. Biochemical and serum analyses were performed using different reagent kits to detect LDH (Le Du, S03034) and CK-MB (Changchun Hui Li, C060) levels in the serum, according to the kit instructions.

### ELISA detection of IL-6, IL-1β, and TNF-α

According to the instructions, different ELISA kits were used to analyze the levels of IL-6, IL-1β, and TNF-α in serum. Additionally, the same method was used to detect IL-6, IL-1β, and TNF-α levels in the cell culture medium. In brief, samples and standard solutions were added to the wells, after which working solution was added and incubated at a constant temperature for 60–90 min. Then, the wells were washed with wash buffer, and chromogenic substrate was added. After being incubated in the dark for 15 min, a stop solution was added. The absorbance of each well was measured at 450 nm, and the concentration of the corresponding samples was calculated based on the standard curve.

### Annexin V-FITC/PI double staining

According to the instructions, cells were digested with trypsin, transferred to centrifuge tubes and centrifuged at 1000 × g at room temperature. The cells were resuspended in 1 × binding buffer and stained with PI and FITC (4 μl/tube) at room temperature for 15 min. Then, 1 × binding buffer (400 μl) was added, and the cells were transferred to flow cytometry tubes. Flow cytometry was performed in the dark, and the data obtained were analyzed using FLOWJO 10.07.

### Dual-labelled immunofluorescence analysis

In brief, H9C2 cells were pretreated with Alda-1, daidzin, followed by LPS treatment. The cells were washed three times with PBS and fixed with an immunofluorescence fixation solution at room temperature for 10 min. After three rinses with PBS for 3 min each, the cells were incubated with an immunofluorescence-permeabilization solution at room temperature for 10 min. Subsequently, the cells were rinsed three times with PBS for 3 min each. The cells were then blocked with an immunofluorescence blocking solution at room temperature for 10 min, followed by three rinses with PBS for 3 min each. The cells were incubated with the primary antibody overnight at 4 °C, followed by three rinses with PBS the next day. Then, the cells were incubated with a fluorescent secondary antibody in the dark for 1 h and stained with DAPI for 10 min. Subsequently, the cells were rinsed with PBS and observed and analyzed in detail using a laser confocal microscope (analyzed using Image-Pro Plus 6.0).

### Western blot analysis

Heart tissues and H9C2 cells were added to RIPA lysis buffer containing phosphatase and protease inhibitors. The heart tissue was ground in an animal tissue grinder at 4 °C at 80 Hz for 10 min, and the supernatant was collected. The cells were sonicated with a cell disruptor at 40 Hz, 2S/cycle, with a 1-s interval, after which they were centrifuged at 4 °C and 10,000 × g for 15 min. Protein quantification was performed using the BCA method. The proteins were denatured by adding 5 × loading buffer and incubation at 100 °C in a metal bath for 10 min, followed by storage at − 80 °C. In order to separate proteins based on their molecular weights, SDS-PAGE technique was employed. The separated proteins were subsequently transferred onto a polyvinylidene difluoride (PVDF) membrane. Following this, the membrane was blocked with 5% skim milk solution at room temperature for 1 h to prevent non-specific binding. Subsequently, the membrane was washed with Tris-buffered saline with Tween-20 (TBST), and primary antibodies were added and incubated overnight at 4 °C to facilitate specific protein detection. On the second day, after the membrane was washed with TBST, secondary antibodies were added and incubated at room temperature for 1.5 h. ECL chemiluminescence was used for imaging. The bands were observed and photographed using a gel imaging system (analyzed using ImageJ-Fiji).

### JC-1 assay

An enhanced mitochondrial membrane potential detection reagent kit was used to measure the mitochondrial membrane potential of H9C2 cells. The specific procedures were performed according to the instructions (analyzed using Image-Pro Plus 6.0).

### ROS analysis

A reactive oxygen species detection kit was used to measure ROS levels in H9C2 cells. The specific procedures were performed according to the instructions (analyzed using Image-Pro Plus 6.0).

### Statistical analysis

Statistical analysis was performed using GraphPad Prism 7 software for intergroup comparisons. The data were reported as mean ± standard deviation. Independent t-test was used for comparisons between two groups. One-way analysis of variance (ANOVA) followed by Tukey's multiple comparison test was used for comparisons among multiple groups as the post hoc analysis. Statistical significance was defined as P < 0.05 (*P < 0.05, **P < 0.005, ^#^P < 0.0001).

## Results

### Activation of ALDH2 improves LPS-induced cardiac dysfunction, alleviates inflammation, and apoptosis

**Fig. 1:**
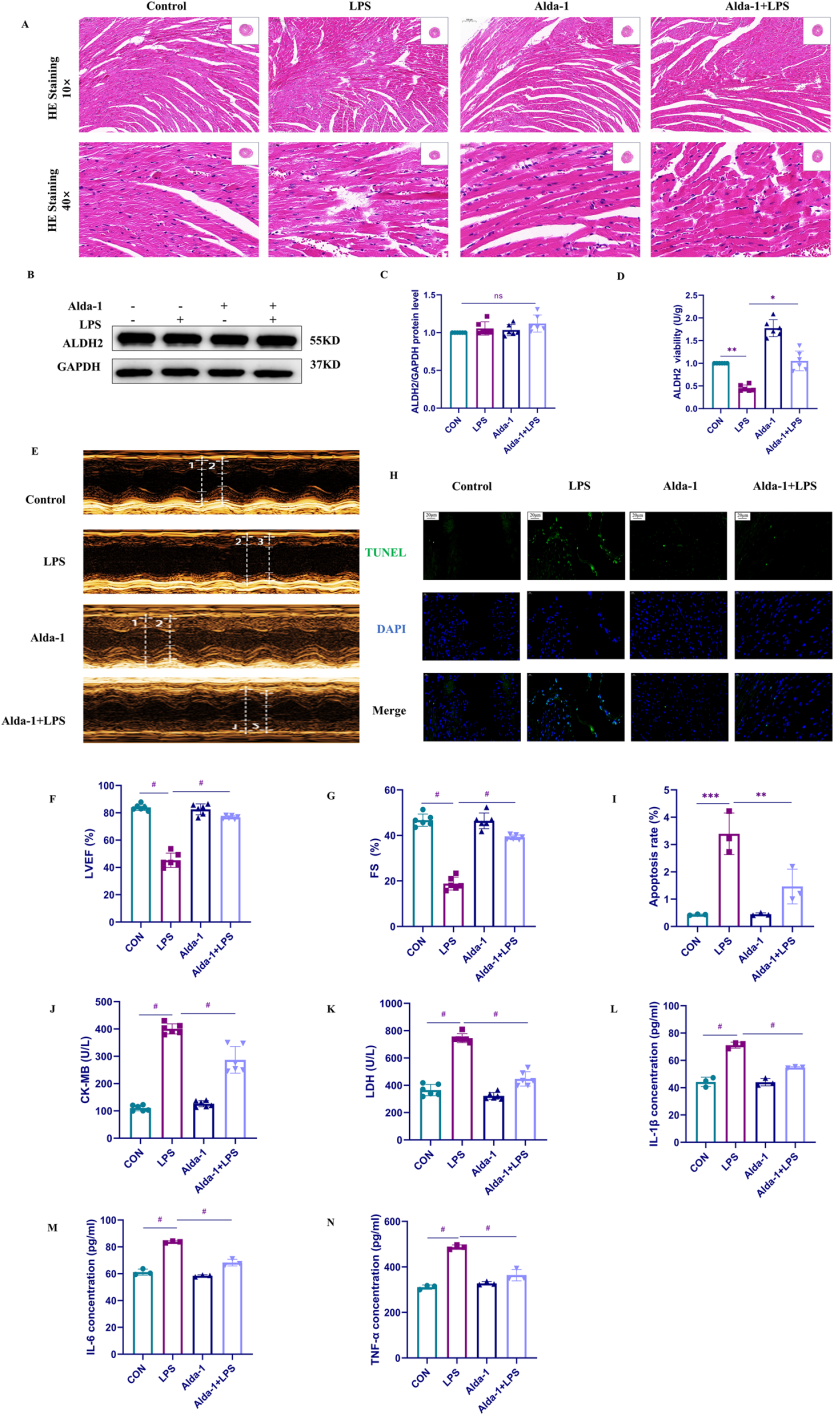
ALDH2 activation improves LPS-induced cardiac dysfunction, inflammation, and apoptosis. **A** Mouse cardiac tissues sections were stained with HE to visualize tissue morphology at a magnification of × 100 and × 400. **B**, **C** ALDH2 protein (from heart tissue homogenate) expression levels were assessed using Western blot analysis, followed by quantitative analysis (n = 6). **D** ALDH2 activity was measured to evaluate its functional status (n = 6). **E** Cardiac function in mice was assessed using color Doppler imaging. **F**, **G** Left ventricular ejection fraction (LVEF) and fractional shortening (FS) were quantitatively analyzed to evaluate cardiac function (n = 6). **H** TUNEL assay was performed to detect myocardial cell apoptosis in mouse cardiac tissue sections at a magnification of × 400. **I** Apoptosis rate (%). **J**, **K** Detection of creatine kins-MB (CKMB) and lactate dehydrogenase (LDH) in the blood of mice (n = 6). **L**–**N** ELISA is employed to detect Interleukin-6 IL-6/IL-1β/TNF-α in serum samples (n = 3)

After administration in mice, we found that the control and Alda-1 groups had stable vital signs, normal diet, and smooth fur, while the LPS group showed obvious lethargy, increased eye discharge, reduced activity, and refusal to eat. Compared with the LPS group, the mice pretreated with Alda-1 showed an improved mental state, no obvious eye discharge, and normal activity and diet after LPS induction. Moreover, the dose of Alda-1 used in the experiment had no obvious toxic and side effects (Additional file 1: Fig S3). We assessed the pathological changes in mouse hearts from different groups using HE staining. In the LPS group, we observed mild edema at the local epicardium of the heart tissue, loose arrangement of connective tissue, myocardial fiber rupture, infiltration of adipocytes or inflammatory cells, and nuclear deformation, while pretreated with Alda-1 reversed these adverse reactions (Fig. 1A). Next, we investigated the changes in ALDH2 expression and activity in mouse hearts after LPS induction. We performed ALDH2 protein expression and activity assays in different groups and made an intriguing observation: neither LPS nor Alda-1 altered the protein level of ALDH2; however, ALDH2 activity significantly changed after LPS treatment. Alda-1 treatment notably elevated ALDH2 activity and reversed the decrease caused by LPS (Fig. 1B–D). Subsequently, we used color Doppler imaging to examine cardiac function in the different groups of mice (Fig. 1E). The results showed that the heart function of mice in the LPS group significantly declined, with FS and LVEF decreasing by approximately 50%, indicating significant damage to cardiac contraction and relaxation. These findings confirmed the successful establishment of the LPS-induced cardiac dysfunction model in mice; Compared with the LPS group, pretreated with Alda-1 significantly improved mouse heart function, with FS and LVEF increasing by approximately 30%. In addition, to demonstrate that ALDH2 can improve LPS-induced myocardial injury, we measured serum cardiac injury markers, including CKMB and LDH, in mice from different groups (Fig. 1F, G). The results showed that compared with those in the control group, the expression levels of CKMB and LDH in the LPS group significantly were increased, while pretreated with Alda-1 significantly reduced their expression. Therefore, activating ALDH2 can protect the heart from LPS-induced injury. Furthermore, we used the TUNEL assay to assess the apoptosis rate in mouse hearts from different groups (Fig. 1H, I), which was increased by LPS induction. However, Alda-1 pretreated attenuated the effect of LPS and significantly reduced apoptosis. In addition, we used ELISA to measure the levels of inflammatory cytokines, including IL-6, IL-1β, and TNF-α, in mouse serum (Fig. 1L–N). The results revealed elevated expression of inflammatory cytokines in the LPS group, which was reversed after Alda-1 pre-treatment. In summary, these results demonstrate that the activation of ALDH2 can improve LPS-induced cardiac dysfunction, alleviate inflammation, and reduce apoptosis.

### Activation of ALDH2 can suppress cGAS/STING signaling pathway in vivo.

**Fig. 2:**
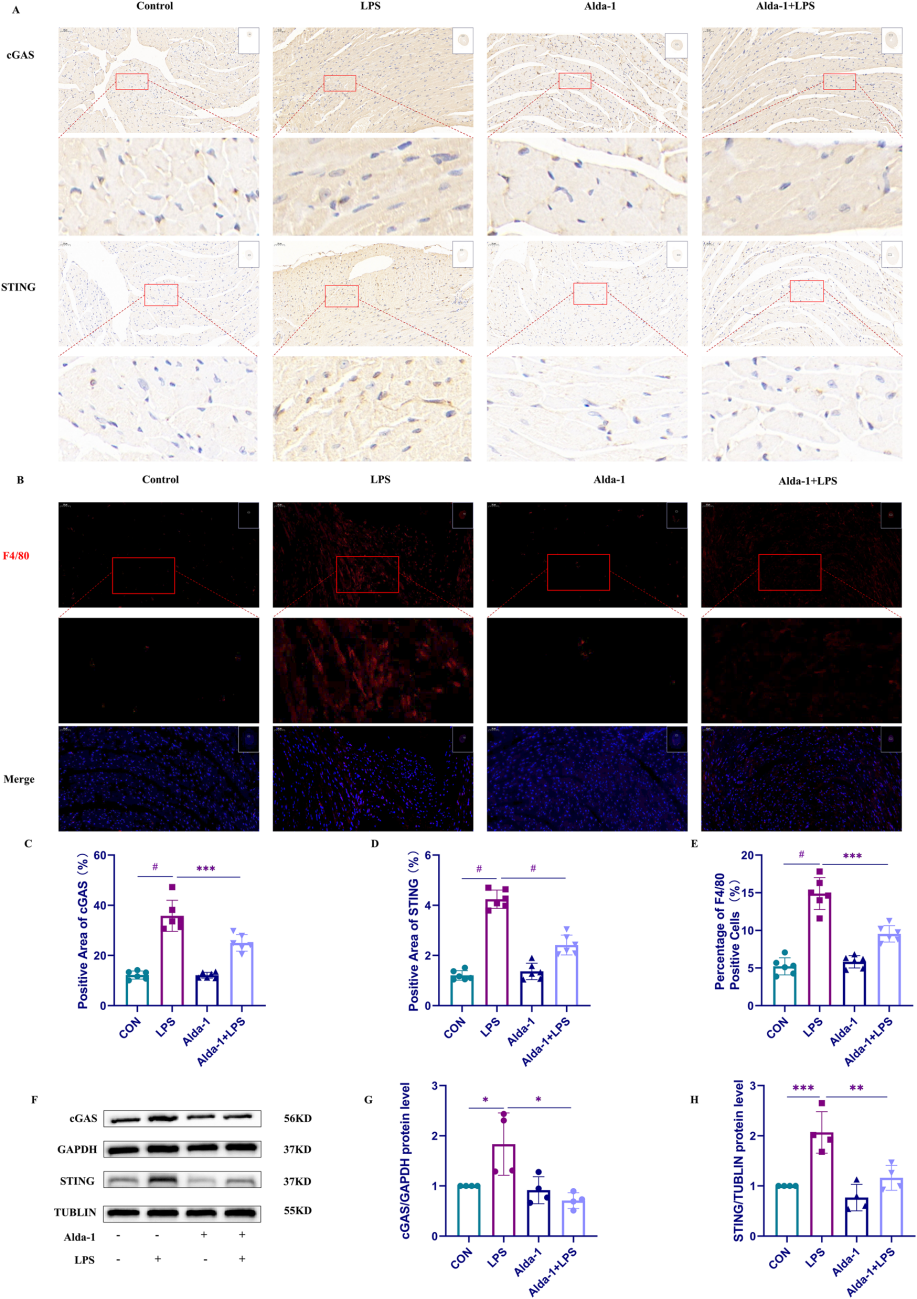
Activation of ALDH2 can suppress cGAS/STING signaling pathway activation induced by LPS in vivo. **A** The expressions of cGAS and STING in cardiac tissues of mice were detected by IHC (magnification = × 200). **B** The expression of F4/80 in the cardiac tissues of mice was detected by IHC (magnification = × 200). **C** Positive area of cGAS (%). **D** Positive area of STING (%). **E** Percentage of F4/80-positive cells (%). **F**–**H** The protein expression of cGAS and STING was detected by Weston blot and quantitative statistics were obtained (n = 4)

We conducted IHC staining to investigate the expression of cGAS and STING in cardiac tissue cells and determine the impact of LPS and Alda-1 on their expression (Fig. 2A, C, D) and performed Western blot analysis (Fig. 2F–H) to assess the protein expression levels of cGAS and STING. We observed an increase in the expression of cGAS and STING in the cardiac tissue of LPS-treated mice compared to the control group by IHC. However, after pretreatment with Alda-1, the expression levels of cGAS and STING significantly decreased. We performed Western blotting to examine the protein expression levels of cGAS and STING to further confirm the relationship between ALDH2 and cGAS/STING in LPS-induced cardiac injury (Fig. 2F–H). The results demonstrated a significant increase in the expression of cGAS and STING proteins following LPS treatment compared to that observed in the control group. However, mice pretreated with Alda-1 exhibited significantly lower cGAS and STING protein expression than the LPS group. We further explored the infiltration of immune cells after LPS-induced myocardial injury. We used immunohistochemical methods to specifically label macrophages (F4/80) and neutrophils (CD14) in mouse heart tissue (Fig. 1B, E and Additional file 1: Fig S2). The results indicate that LPS caused an increase in neutrophils and macrophages in the myocardial tissue, and Alda-1 was able to reverse this outcome. These findings align with our previous hypothesis that ALDH2 may exert a protective effect in LPS-induced cardiac injury through the cGAS/STING pathway.

### ALDH2 protected H9C2 cells from LPS-induced cell apoptosis and inflammation in vitro

To further investigate the mechanisms of ALDH2 and cGAS/STING in SIC, we conducted a more in-depth study in H9C2 cells. Firstly, we screened the drug concentrations of LPS, Alda-1, and daidzin in the cells. Detailed results can be found in Additional file 1: Fig S1.

**Fig. 3:**
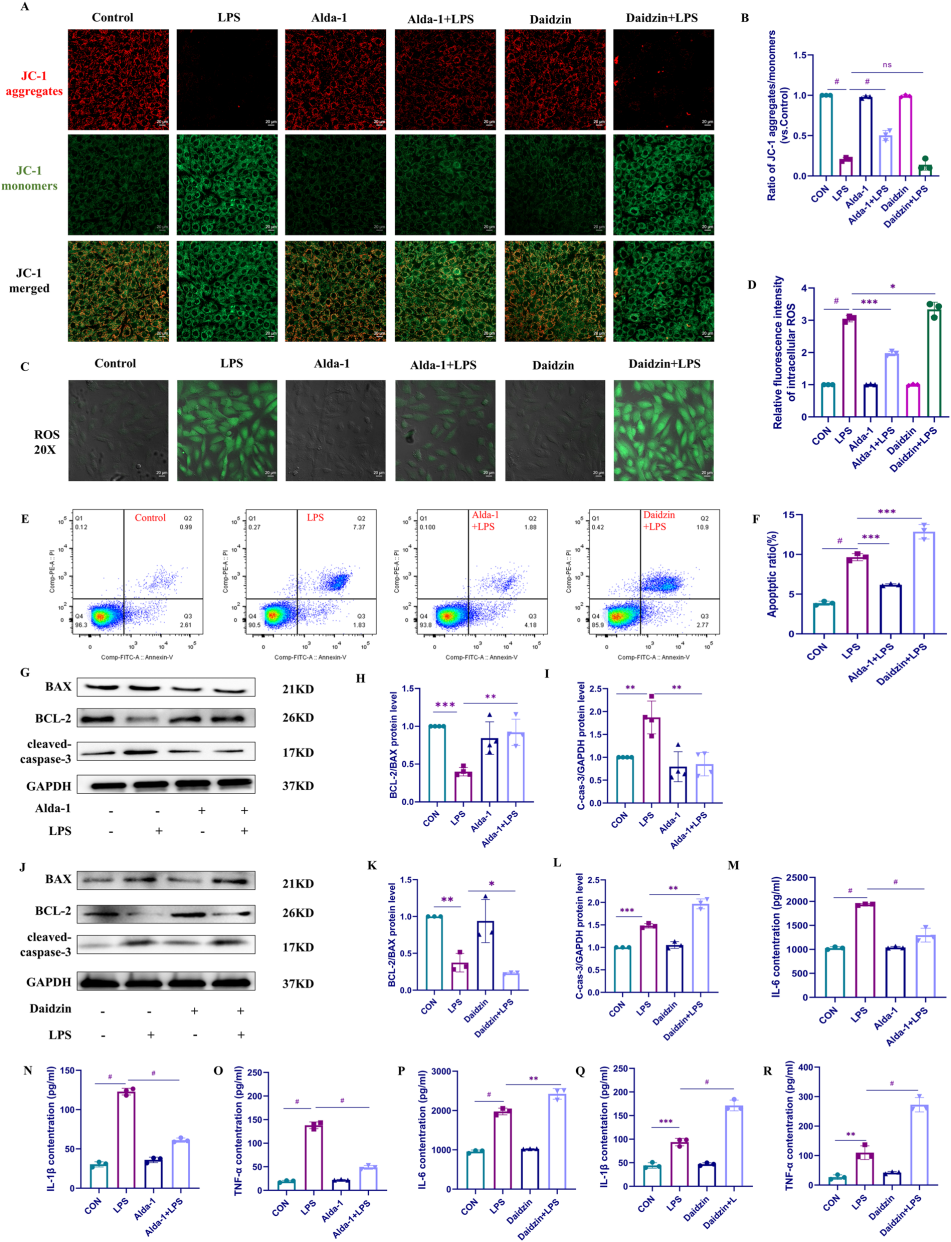
ALDH2 protected H9C2 cells from LPS-induced cell apoptosis and inflammation. **A** Mitochondrial membrane permeability was assessed by staining H9C2 cells from different groups with JC-1 dye, followed by evaluation using confocal microscopy. **B** Ratio of aggregates/monomers (vs. Control). **C** ROS levels were evaluated by staining H9C2 cells with DCFH-DA dye, followed by assessment using confocal microscopy. **D** Relative fluorescence intensity of intracellular ROS. **E**, **F** Apoptosis rates of H9C2 cells from different groups were measured and quantitatively analyzed using flow cytometry (n = 3). **G**–**I** The expression levels of BAX/BCL-2/cleaved caspase-3 proteins were evaluated by Western blot analysis after Alda-1 pretreatment and LPS stimulation in H9C2 cells (n = 4). **J**–**L** The expression levels of BAX/BCL-2/cleaved caspase-3 proteins were evaluated by Western blot analysis after Alda-1 pretreatment and LPS stimulation in H9C2 cells (n = 3). **M**–**O** The expression levels of IL-6, IL-1β, and TNF-α were detected by ELISA after Alda-1 pretreatment and LPS stimulation in H9C2 cells (n = 3). **P**–**R** The expression levels of IL-6, IL-1β, and TNF-α were detected by ELISA after daidzin pretreatment and LPS stimulation in H9C2 cells (n = 3)

The effect of ALDH2 on early apoptosis in cardiac cells was examined by measuring mitochondrial membrane permeability (Fig. 3A, B). Consistent with our hypothesis, H9C2 cells exhibited significantly higher mitochondrial membrane permeability after LPS stimulation than those in the control group. However, the pretreatment of H9C2 cells with the ALDH2 activator Alda-1 attenuated the effect of LPS, resulting in a significant decrease in mitochondrial membrane permeability. The opposite effect was observed when H9C2 cells were pretreated with an ALDH2 inhibitor daidzin; the daidzin synergistically increased the mitochondrial membrane permeability of H9C2 cells. Considering the role of oxidative stress in SIC, we measured the levels of ROS in H9C2 cells from different groups using DCFH-DA staining (Fig. 3C, D). Our findings demonstrated that LPS stimulation significantly increased ROS production in H9C2 cells. However, pretreatment of H9C2 cells with Alda-1 reduced the stimulatory effect of LPS, significantly decreasing ROS levels. Interestingly, in the case of daidzin pretreatment, we observed a synergistic effect with LPS, resulting in an increase in ROS levels in H9C2 cells. We used Western blotting to further investigate the role of ALDH2 in LPS-induced H9C2 cells and quantitatively analyzed apoptosis-related proteins in H9C2 cells in the different groups (Fig. 3G–L). The results revealed a significant increase in proapoptotic protein BAX and cleaved caspase-3 and a decrease in anti-apoptotic protein BCL-2 after LPS treatment. However, in the Alda-1 pretreatment group, a reversal of these effects was observed. Interestingly, daidzin exhibited a synergistic effect with LPS, amplifying the observed changes. Furthermore, we conducted flow cytometry analysis to measure the apoptosis rate of H9C2 cells from different groups, as depicted in Fig. 3E, F. The results were consistent with those obtained in our previous experiments. LPS stimulation significantly increased the apoptosis rate of H9C2 cells. However, pretreatment of H9C2 cells with Alda-1 reversed the high apoptosis rate induced by LPS. Intriguingly, daidzin exhibited a synergistic effect with LPS, leading to a further increase in the apoptosis rate compared to that achieved with LPS alone. Meanwhile, we employed the ELISA method to detect IL-6/IL-1β/TNF-α in the supernatant of cell culture media from each group. We observed that pre-treatment of H9C2 cells with Alda-1 led to a reduction in the expression of inflammatory factors induced by LPS (Fig. 3M–O). Conversely, pre-treatment with daidzin synergistically exacerbated the detrimental effects of LPS, resulting in higher expression of inflammatory factors (Fig. 3P–R). These findings collectively suggest the involvement of ALDH2 in the damage caused by LPS-induced H9C2 cells.

### ALDH2 inhibited cGAS/STING signaling pathway in LPS-stimulated H9C2 cells

**Fig. 4:**
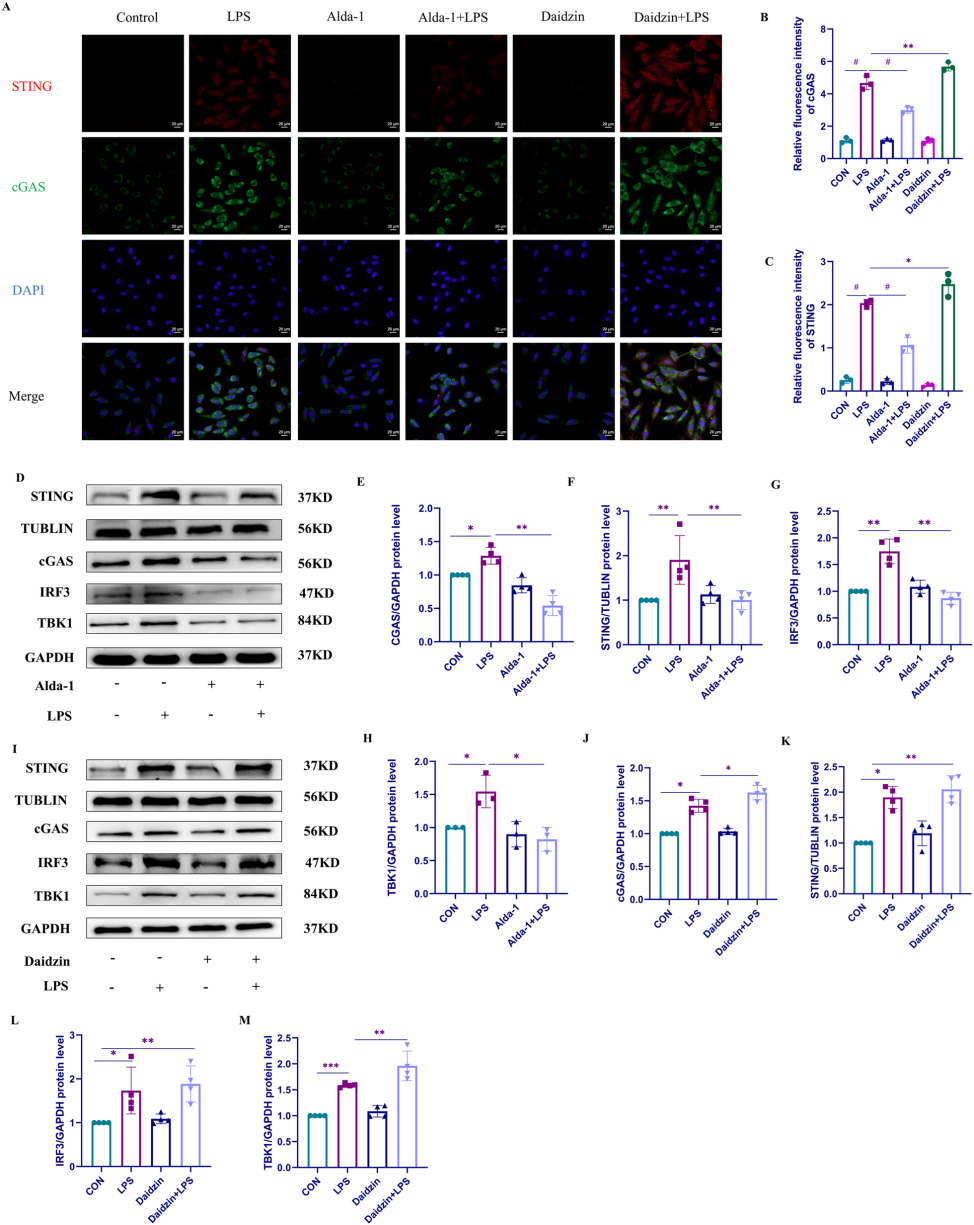
ALDH2 inhibited cGAS/STING signaling pathway in LPS-stimulated H9C2 cells. **A** The effects of Alda-1 and daidzin on the levels of cGAS and STING in LPS-induced H9C2 cells were observed using cell immunofluorescence assay. **B** Relative fluorescence intensity of cGAS. **C** Relative fluorescence intensity of STING. **D**–**H** The expression levels of cGAS and its downstream proteins STING, IRF3, and TBK1 in H9C2 cells pretreated with Alda-1 and stimulated with LPS were detected and quantitatively analyzed using Western blot analysis (n = 4 or n = 3). **I**–**M** The expression levels of cGAS and its downstream proteins STING, IRF3, and TBK1 in H9C2 cells pretreated with daidzin and stimulated with LPS were detected and quantitatively analyzed using Western blot analysis (n = 4)

We first used cell immunofluorescence to observe the localization and expression of cGAS and STING in H9C2 cells and investigate the role of ALDH2 in H9C2 cells (Fig. 4A–C). Our results revealed that both proteins were primarily expressed in the cytoplasm, consistent with cGAS being a cytoplasmic sensor. LPS stimulation significantly increased the expression of cGAS and STING in H9C2 cells. However, when we pretreated the cells with Alda-1, the effect of LPS was attenuated, and the protein expression levels of cGAS and STING in H9C2 cells were significantly reduced compared to those in the LPS group. To further investigate the protein expression and quantitatively analyze the cGAS/STING signaling pathway, we pretreated H9C2 cells with Alda-1 for 2 h, followed by LPS stimulation. We then detected the expression of cGAS/STING pathway-related proteins in H9C2 cells using Western blotting (Fig. 4D–H). Our findings demonstrated that the expression of cGAS significantly increased after LPS treatment, and its downstream proteins, including STING, IRF3, and TBK1, exhibited similar trend changes. In the Alda-1 pretreatment group, the expression levels of cGAS, STING, IRF3, and TBK1 were significantly lower than those in the LPS group, consistent with the results of the cell immunofluorescence analysis. To further investigate whether ALDH2 exerts its effects through the cGAS/STING pathway, we pretreated H9C2 cells with the ALDH2 inhibitor daidzin. After 2 h, we stimulated the cells with LPS. Similarly, we used Western blotting to detect the expression of cGAS/STING signaling pathway-related proteins (Fig. 4I–M). Our results were consistent with the cell immunofluorescence findings, showing a synergistic effect of daidzin with LPS. We observed increased expression of cGAS and similar trend changes in its downstream proteins, including STING, IRF3, and TBK1.The results obtained from cell immunofluorescence and these small molecule treatments collectively demonstrate the involvement of ALDH2 in regulating the cGAS/STING signaling pathway in LPS-stimulated H9C2 cells.

### si-cGAS attenuated apoptosis and inflammation induced by LPS and daidzin through the inhibition of STING signaling pathway and the production of ROS level

**Fig. 5:**
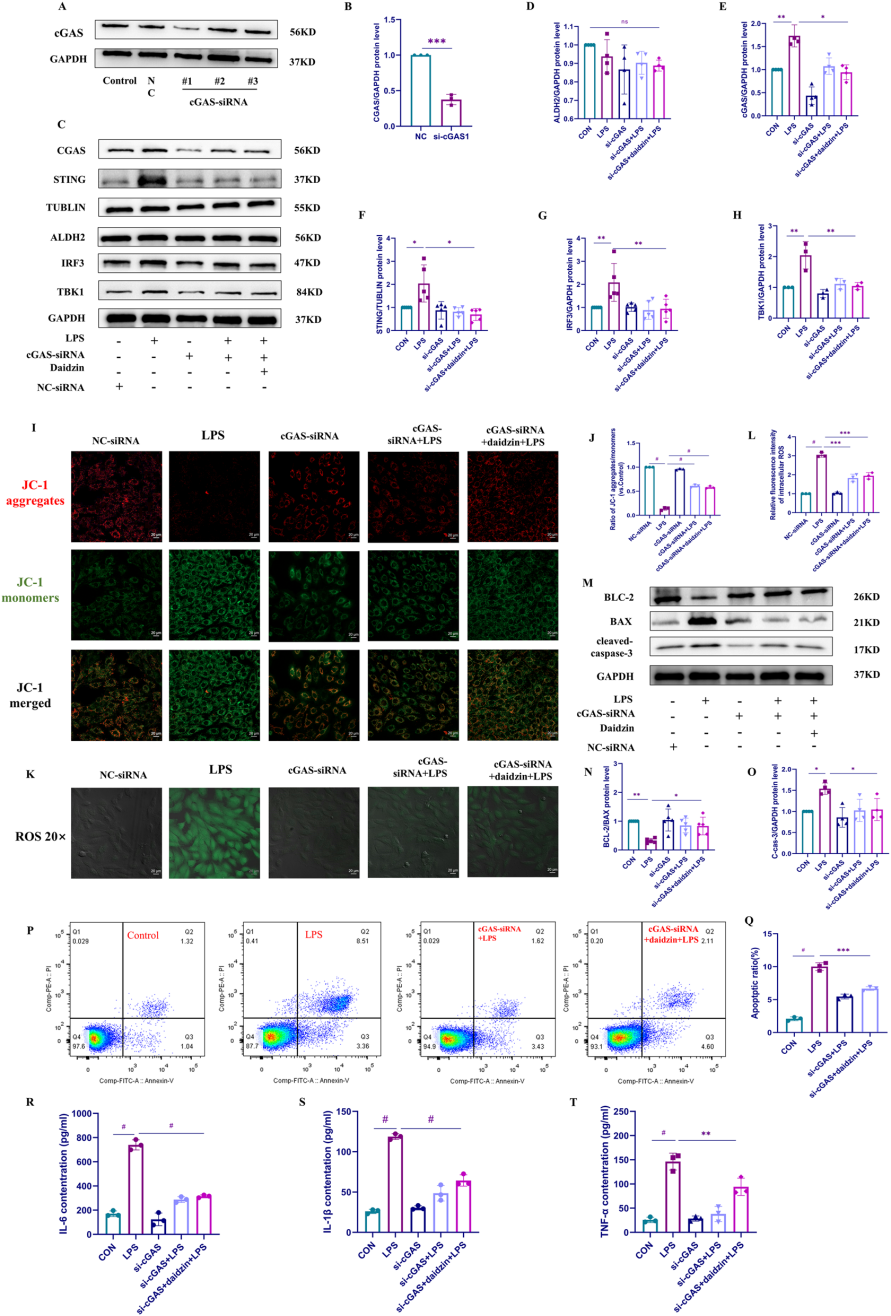
ALDH2 involvement in regulating the cGAS/STING signaling pathway in LPS-stimulated H9C2 cells. **A**, **B** Western blot analysis was performed to screen cGAS-siRNA and quantify its expression levels (n = 3). **C**–**H** Western blot analysis was conducted to detect the protein expression levels of cGAS, STING, IRF3, TBK1, and ALDH2 in H9C2 cell lysates, followed by quantitative analysis (n = 3 or n = 4 or n = 5). **I** Mitochondrial membrane potential permeability was evaluated by staining H9C2 cells with JC-1 dye and analyzing them using confocal microscopy. **J** Ratio of JC-1 aggregates/monomers (vs. Control). **K** ROS levels were assessed by staining H9C2 cells with DCFH-DA dye and evaluating them using confocal microscopy. **L** Relative fluorescence intensity of intracellular ROS. **M**–**O** Western blot analysis was used to analyze the protein expression levels of BAX, BCL-2, and cleaved caspase-3 in H9C2 cell lysates, followed by quantitative analysis (n = 4 or n = 5). **P**, **Q** Flow cytometry analysis was performed to analyze the apoptosis rate of H9C2 cells (n = 3). **R**–**T** ELISA was used to evaluate the expression levels of IL-6, IL-1β, and TNF-α in the supernatant of H9C2 cells after cGAS knockdown in different groups (n = 3)

We used siRNA to silence cGAS and validate our previous hypothesis, (Fig. 5A, B). We aimed to determine whether ALDH2 could still alleviate inflammation, apoptosis, and oxidative stress in LPS-induced H9C2 cells. We used Western blotting to verify the changes in proteins in the ALDH2 and cGAS/STING signaling pathways (Fig. 5C–H). Our results indicated that knocking down cGAS did not affect the protein expression level of ALDH2. Moreover, the protein expression levels of STING, IRF3, and TBK1 downstream of cGAS remained unchanged. However, in LPS-stimulated H9C2 cells, knocking down cGAS decreased the protein expression levels of cGAS, STING, IRF3, and TBK1. Additionally, daidzin pretreatment did not significantly increase the aforementioned protein expression levels. To further investigate the impact of cGAS silencing, we evaluated mitochondrial membrane potential in cGAS-silenced H9C2 cells (Fig. 5I, J). Consistent with the Western blot analysis, knocking down cGAS, LPS, and daidzin did not increase mitochondrial membrane potential permeability as observed previously. Additionally, Western blotting revealed that knocking down cGAS attenuated the apoptotic effect of LPS stimulation on H9C2 cells, as evidenced by decreased protein expression levels of BAX and cleaved caspase-3. Notably, daidzin did not synergistically increase their expression (Fig. 5M–O). Subsequently, we confirmed this phenomenon using flow cytometry analysis (Fig. 5P, Q). Our findings revealed that with cGAS silencing, LPS and daidzin failed to induce a high apoptosis rate in H9C2 cells. This finding indicates that cGAS activation by LPS increases apoptosis in H9C2 cells, and ALDH2 exerts its anti-apoptotic effect through cGAS. Similarly, we measured the levels of reactive ROS in cGAS-silenced H9C2 cells and observed that LPS did not stimulate ROS production in H9C2 cells after cGAS knockdown. Furthermore, the effect of daidzin on ROS levels was weakened (Fig. 5K, L). Since inflammation plays a crucial role in cardiovascular diseases, we further utilized ELISA to measure the levels of IL-6, IL-1β, and TNF-α in the supernatant of cells after cGAS knockdown (Fig. 5R–T). Consistent with the previous experiments, knocking down cGAS did not induce a significant increase in the production of inflammatory factors in H9C2 cells stimulated by LPS. Additionally, the inhibitory effect of daidzin was also observed. These results further confirm that cGAS is an important target for the actions of LPS and ALDH2.

## Discussion

**Fig. 6:**
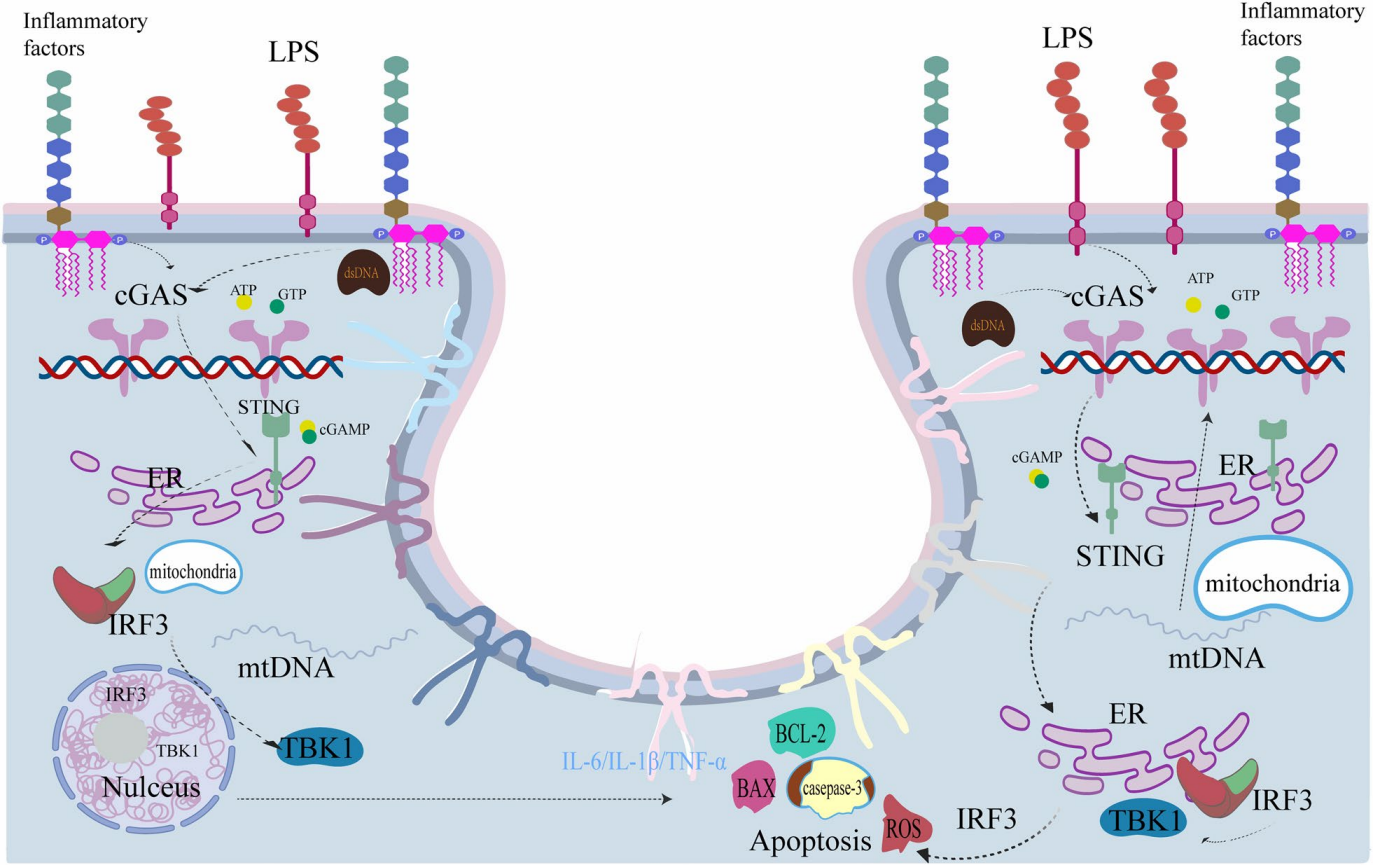
The working model of LPS in the cGAS/STING signaling pathway. LPS increases apoptosis, inflammation, and oxidative stress through targeting the cGAS/STING signaling pathway

Sepsis is a life-threatening disease caused by a dysregulated response to infection, leading to the development of multiple organ dysfunction syndrome (Fernando et al. 2018). Studies have shown that most patients with sepsis have accompanying cardiac dysfunction, which is closely associated with mitochondria, but the underlying mechanisms are unclear (Li et al. 2020; Wang et al. 2022). In this study, we used LPS to simulate the occurrence and development of SIC and explored the pathological mechanisms. After LPS stimulation in mice or H9C2 cells, we observed cardiac dysfunction, elevated myocardial injury markers, increased expression of inflammatory factors and ROS, and significantly increased apoptosis in cardiomyocytes. However, when ALDH2 activity was increased, the adverse effects of LPS stimulation were reversed. Furthermore, we investigated the mechanism of ALDH2 in LPS-induced cardiac injury by using siRNA-mediated knockdown of cGAS. Interestingly, after siRNA-mediated knockdown of cGAS, the effects of ALDH2 and LPS were significantly attenuated. Although the detailed pathogenesis of SIC exceeds the scope of our current experimental study, our data confirm our previous hypothesis that ALDH2 reduces mitochondrial damage, inflammation, and apoptosis through the cGAS/STING signaling pathway, thereby protecting against LPS-induced cardiac injury (Fig. 6). This finding suggests that ALDH2 and cGAS/STING have great potential in the prevention and treatment of SIC.

ALDH2 is a mitochondrial enzyme that protects cells from damage caused by aldehydes such as acetaldehyde or lipid-derived aldehydes such as 4-HNE (Cao et al. 2022). Alda-1 is a specific small molecule activator of ALDH2 that prevents the generation of toxic aldehydes (Sidramagowda Patil et al. 2020). Previous studies have shown that ALDH2 protects against CVDs, and our study further supports this finding. Our results revealed that pretreatment with Alda-1 significantly improved cardiac dysfunction induced by LPS, whereas pretreatment with daidzin, a specific small molecule inhibitor of ALDH2, synergistically worsened myocardial injury in conjunction with LPS. Interestingly, both LPS and Alda-1 or daidzin alone altered the activity of ALDH2 without affecting its protein expression, which was consistent with previous research findings (Hu et al. 2015). Previous studies have shown that ALDH2 can exert cardioprotective effects by regulating autophagy and altering mitochondrial membrane potential (Wang et al. 2020). Similarly, in our study, we examined inflammation and mitochondrial membrane potential. Consistent with previous research, an increase in ALDH2 activity reversed the high levels of inflammatory factors and increased mitochondrial membrane permeability induced by LPS stimulation. These findings suggest that ALDH2 can protect the myocardium in SIC by reducing inflammation and early apoptosis in cardiomyocytes. Therefore, our findings suggest that ALDH2 activity may play a key role in the prevention and treatment of SIC.

Although previous studies (Boyd et al. 2006; Wang et al. 2022; Lim and Staudt 2013) have mostly focused on the relationship and roles of extracellular stimuli and nuclear membrane receptors, our study revealed that the cytoplasmic DNA sensor cGAS and its downstream effector STING played significant roles in LPS-induced cardiac injury. The cGAS/STING pathway is an important signaling pathway involved in the immune response, and excessive pathway activation can lead to inflammation or the development of autoimmune diseases (Gui et al. 2019). When we used siRNA to knock down cGAS, the inflammation, apoptosis, and excessive ROS induced by LPS were improved. Even when we pretreated the cells with daidzin to reduce the activity of ALDH2, the synergistic effect between LPS and daidzin was not observed, rendering the damaging effects of LPS and daidzin ineffective. These findings lead us to consider that since DNA is typically located in the nucleus and mitochondria, when mitochondrial integrity is compromised, mtDNA accumulates in the cytoplasm and triggers the production of the second messenger cGAMP, which then binds and activates the adaptor protein STING, recruiting TBK1 and proinflammatory cytokines, disrupting ATP supply and mitochondrial bioenergetics, and resulting in irreversible cell death (Li et al. 2022; Gui et al. 2019; Wang et al. 2020; Zhao et al. 2018). In previous studies, we often overlooked the excessive accumulation and release of dsDNA and mtDNA as danger-associated molecules when the organism is injured. Surprisingly, some studies have shown that the expression of cGAS/STING is inhibited when mtDNA and proinflammatory factors accumulate to a certain extent in an organism, possibly due to the excessive consumption of the cytoplasmic DNA sensor cGAS, and inflammation persists, leading to this result (Wang et al. 2020). However, in our study, the LPS concentration used to simulate SIC did not result in this effect. We hypothesize that ALDH2 can reduce the accumulation of inflammatory factors or mtDNA through other pathways, thereby preventing their entry into the cytoplasm. Alternatively, mainstream research on cGAS/STING has been conducted in immune cells, and there may be different changes in cardiomyocytes. To this end, we also conducted some exploration of macrophages and neutrophils in heart tissue by immunohistochemical staining, and found that activation of ALDH2 can also restrict the activation of macrophages and neutrophils, which may also be a way to reduce cardiac inflammation, but this has not been deeply explored in our in vivo experiments. The relevant literature has reported that when non-ischemic injury occurs (Ninh and Brown 2021), cardiomyocytes are the initiators of cardiac inflammation and may largely determine adverse remodeling and the progression of heart failure by coordinating leukocyte recruitment (Suetomi et al. 2019, 2018). Moreover, studies have shown that cardiomyocytes are direct and effective sources of cardiac inflammation, directly initiating the occurrence of the heart, and when myocardial damage occurs, the cGAS/STING pathway, can result in transcriptional activation of interferon regulatory factors and other chemoattractants important in the recruitment of leukocytes (Hu et al. 2020). Although the extent to which cardiomyocytes participate in inflammation has not been well understood, our in vitro experiments have verified some contributions of cardiomyocytes to inflammation, which is consistent with previous studies (Suetomi et al. 2019; Rodríguez-Nuevo and Zorzano 2019; Zhang et al. 2015). In future studies, exploring the respective contributions of cardiomyocytes and immune cells to cardiac inflammation is also the focus of our research, because it is also an important entry point for the exploration of SIC mechanism.

This report presents several questions and evidence. Inflammation, apoptosis, and mitochondrial damage are important features of SIC (Zheng et al. 2017; Zhong et al. 2019; Pang et al. 2019) and are currently recognized mechanisms of SIC. This study used pharmacological methods to enhance or inhibit the activity of ALDH2 to observe its role in LPS-induced cardiac injury. The results demonstrated that ALDH2 could reverse myocardial dysfunction, inflammation, and apoptosis induced by LPS. Furthermore, using siRNA-mediated knockdown of cGAS to inhibit the cGAS/STING signaling pathway, it was found that the beneficial effects of ALDH2 on LPS-induced cardiac injury could be counteracted. Importantly, we demonstrated the crucial role of ALDH2 and cytoplasmic DNA sensors in LPS-induced cardiac injury. Although it is premature to consider its clinical application value, these findings suggest that in future work, we can focus on the mechanisms of mitochondria and cytoplasm to provide important evidence for further research on SIC.

## Conclusion

ALDH2 alleviated LPS-induced cardiac dysfunction, inflammation, and apoptosis through inhibited the cGAS/ STING pathway, thereby protecting against SIC.

### Supplementary Information

**Additional file 1.** **Figure S1.** Dose screening of LPS, Alda-1, and daidzin in H9C2 cells. **Figure S2.** Alda-1 can reverse the infiltration of neutrophils in myocardial tissue after LPS stimulation. **Figure S3.** Alda-1 was injected intraperitoneally to detect liver and renal toxicity at 7 and 14 days. **Supplementary Table 1.** The details of the drugs and related reagents used in this study are presented in Table 1.

## Data Availability

The datasets of the current study are available from the corresponding author on reasonable request.

## Abbreviations

SIC: Sepsis-induced cardiomyopathy
CVDs: Cardiovascular diseases
ALDH2: Aldehyde dehydrogenase 2
cGAS: Cyclic GMP-AMP synthase
STING: Stimulator of interferon genes
LPS: Lipopolysaccharide
DAMP: Damage-associated molecular pattern
ROS: Reactive oxygen species
mtDNA: Mitochondrial DNA
dsDNA: Double-stranded DNA
LVEF: Left ventricular ejection fraction
FS: Fractional shortening
siRNA: Small interfering RNA
CKMB: Creatine kinase-MB
LDH: Lactate dehydrogenase
BAX: BCL2-associated X
BCL-2: B-cell lymphoma-2
TBK1: TANK- binding kinase 1
IRF3: Interferon regulatory factor 3
IL-6: Interleukin-6
IL-1β: Interleukin-1 beta
TNF-α: Tumor necrosis factor alpha
HE: Hematoxylin–eosin staining
IHC: Immunohistochemistry
TUNEL: Terminal deoxynucleotidyl transferase dUTP nick-end labeling
IF: Immunofluorescence
WB: Western blot
ELISA: Enzyme-linked immunosorbent assay
PBS: Phosphate-buffered saline
PVDF: Polyvinylidene difluoride
TBST: Tris-buffered saline with Tween-20
ANOVA: One-way analysis of variance
