# Supplementary material for: ALDH2 mitigates LPS-induced cardiac dysfunction, inflammation, and apoptosis through the cGAS/STING pathway
Source: Mol Med. 2023 Dec 20;29:171. doi: 10.1186/s10020-023-00769-5 (PMC10731778; doi:10.1186/s10020-023-00769-5)
Supplement: Supplementary file 1 — Additional file 1. Figure S1. Dose screening of LPS, Alda-1, and daidzin in H9C2 cells. Figure S2. Alda-1 can reverse the infiltration of neutrophils in myocardial tissue after LPS stimulation. Figure S3. Alda-1 was injected intraperitoneally to detect liver and renal toxicity at 7 and 14 days. Supplementary Table 1. The details of the drugs and related reagents used in this study are presented in Table 1. [file 10020_2023_769_MOESM1_ESM.docx]

**Supplementary materials**

**ALDH2 mitigates LPS-induced cardiac dysfunction, inflammation, and apoptosis through the cGAS/STING pathway.**

Haoran Liu^1,2†^, Qin Hu^1,2^, Ke Ren^4^, Pengxin Wu^4^, Yang Wang^4*^, Chuanzhu Lv^2,3,4*^

**Supplementary Figures:**

**Figure S1. Dose screening of LPS, Alda-1, and daidzin in H9C2 cells.**

(A-C) Western blot to detect the protein expression of ALDH2 and cGAS in H9C2 cells stimulated with different concentrations of LPS, followed by quantitative analysis. (D) The effect of Alda-1 at 0/5/10/20/30/60/120μM on H9C2 cell viability was assessed. (E) The effect of daidzin at 0/5/10/30/60/120/240/480μM on H9C2 cell viability was assessed.

**
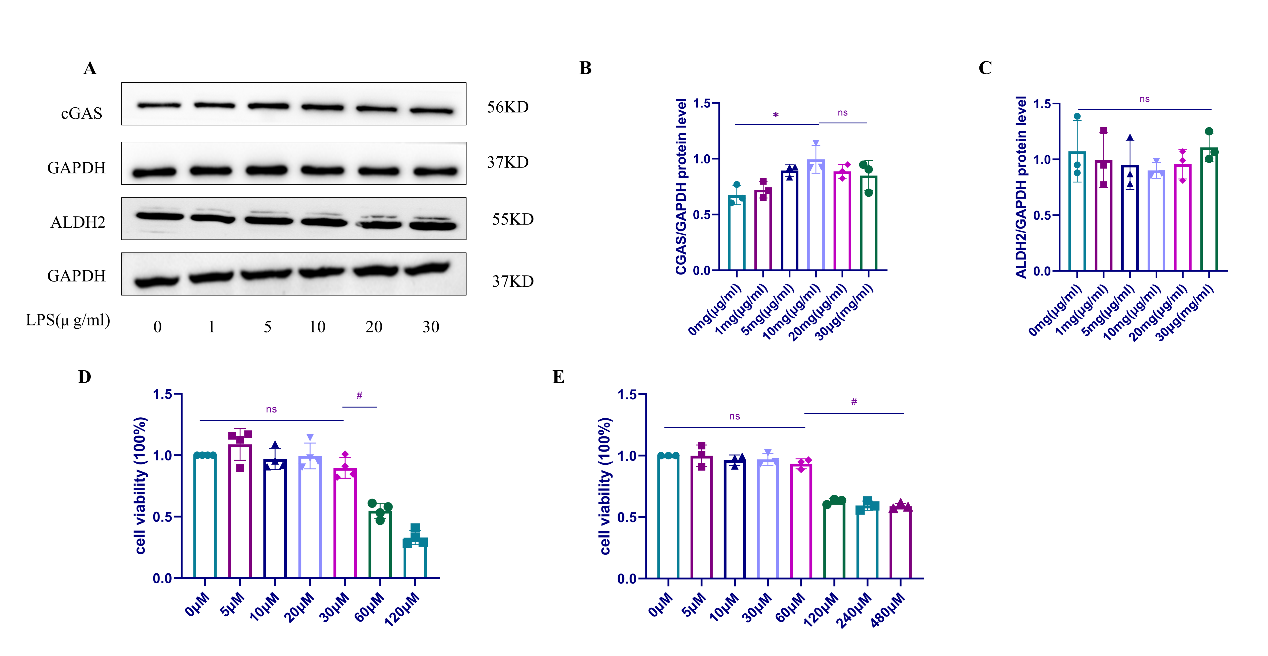
**

**Figure S2. Alda-1 can reverse the infiltration of neutrophils in myocardial tissue after LPS stimulation.**

The expression of CD14 in the cardiac tissues of mice was detected by IHC (magnification = ×200).

**
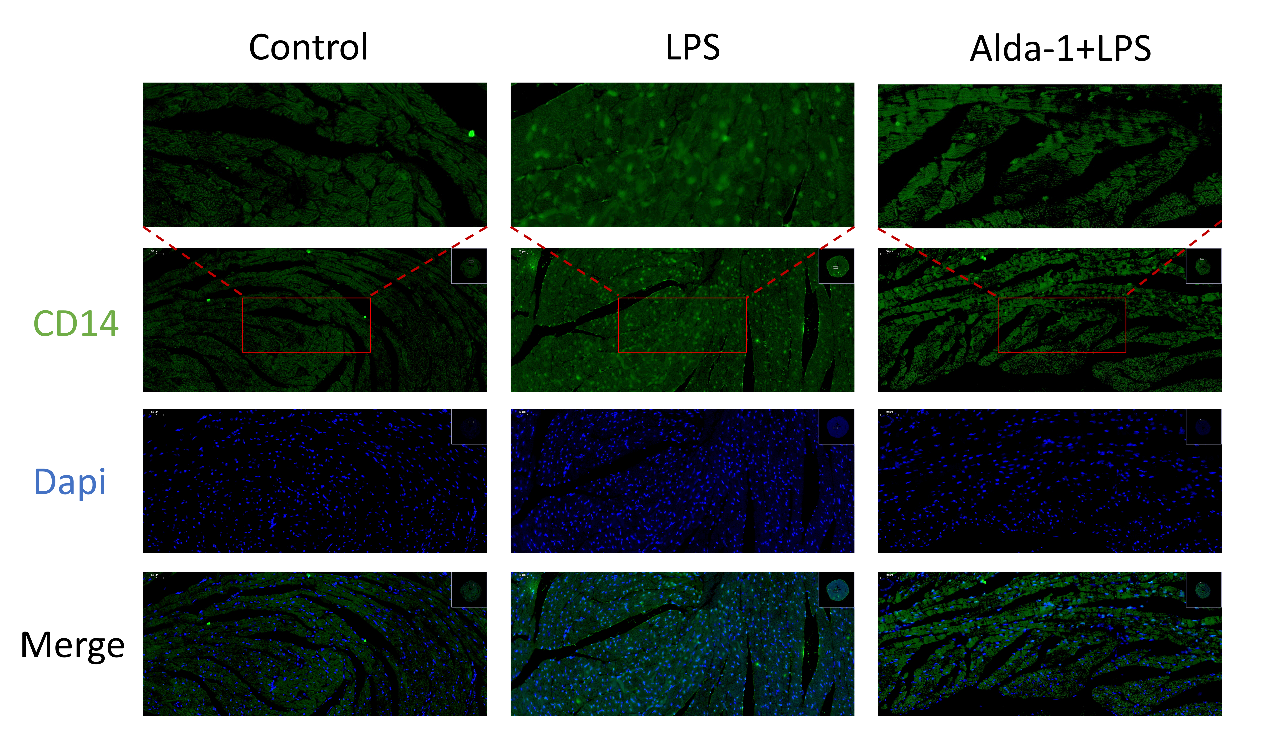
**

**Figure S3. Alda-1 was injected intraperitoneally to detect liver and renal toxicity at 7 and 14 days**

HE staining was used to analyze the pathological changes of liver and kidney after 7 and 14 days of Alda-1 treatment.

**
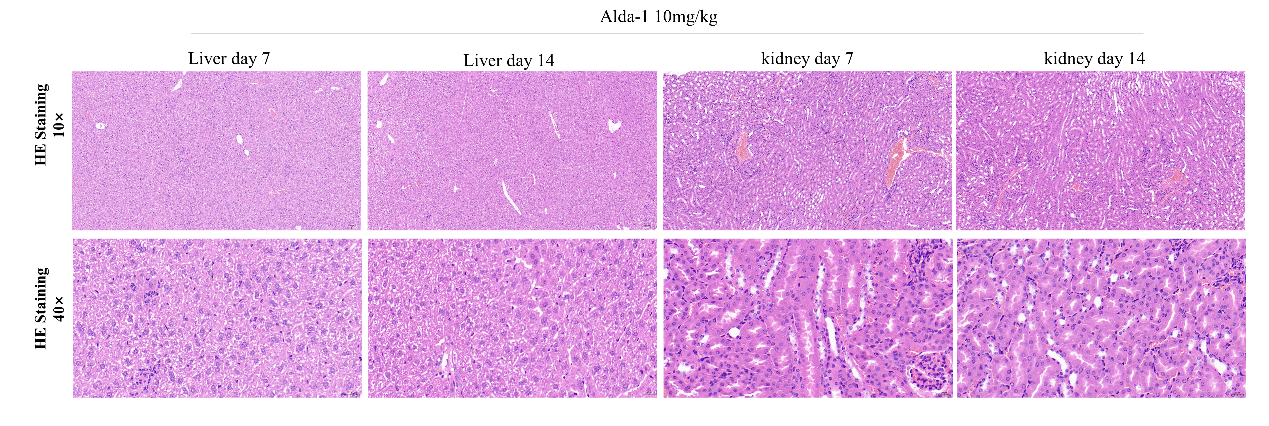
**

**Supplementary Table 1**

The details of the drugs and related reagents used in this study are presented in Table 1.

| Drugs/Reagents | Manufacturer | Catalog Number | Usage /Dosage |
| --- | --- | --- | --- |
|  |  |  |  |
| Fetal bovine serum | Gibco | 30044333 |  |
| DMEM culture medium | Gibco | 8123234 |  |
| Opti-MEM™ I | Gibco | 31985070 |  |
| PBS | Gibco | 8123157 |  |
| FITC Annexin V Apoptosis Detection Kit | BD | 2119964 |  |
| Primary and secondary antibody dilution solution | BOSTER | 17K28C17 |  |
| Alda-1 | Selleck | S5800 | 20uM |
| Daidzin | Selleck | S2289 | 48uM |
| LPS | Sigma | L3129-10MG | 10ug/ml(vitro);10mg/kg(vivo) |
| Color Pre-stained Protein Marker | Thermo | 26616 |  |
| Lipofectamine 3000 | Invitrogen | L3000008 |  |
| siRNA-cGAS | HanHeng Biology | RNA-cGAS-si4(Sense strand:  GAAUCGAGCUAGAAGAAUATT; antisense strand: UAUUCUUCUAGCUCGAUUCTT) |  |
| TNF-α, IL-6, IL-1β Rat ELISA Kit | RayBiotech | RX202412M, RX203049M, RX203063M |  |
| TNF-α, IL-6, IL-1β Mouse ELISA Kit | Solarbio | SEKM-0034, SEKM-0007, SEKM-0002 |  |
| Pierce™ Rapid Gold BCA | Thermofisher | A53225 |  |
| ALDH2 Activity Assay Kit | Solarbio | BC5515 |  |
| Cell Counting Kit-8 | Dojindo | CK04 |  |
| Reactive Oxygen Species Assay Kit | Beyotime Biotechnology | S0033S |  |
| Enhanced mitochondrial membrane potential assay kit with JC-1 | Beyotime Biotechnology | C2003S |  |
| Anti-ALDH2 | HUABIO | M1509-1 | 1:1000 |
| Anti-cGAS | HUABIO | HA500023 | 1:1000 |
| Anti-STING | Abcam | ab288157 | 1:1000 |
| Anti-IRF3 | HUABIO | SD2062 | 1:1000 |
| Anti-TBK1 | HUABIO | HA601045 | 1:1000 |
| Anti-Caspase-3 | Abcam | ab184787 | 1:2000 |
| Anti-Bax Antibody | Abcan | ab32503 | 1:1000 |
| Anti-BCL-2 | HUABIO | ET1702-53 | 1:1000 |
| Anti-GAPDH | HUABIO | ET1601-4 | 1:50000 |
| Anti-beta Tubilin | HUABIO | ET1602-4 | 1:20000 |
| iFluroTM488 Conjugated Goat anti-rabbit IgG | HUABIO | HA1121 | 1:500 |
| iFluroTM594 Conjugated Goat anti-mouse IgG | HUABIO | HA1121 | 1:500 |
| HRP Conjugated Goat anti-mouse IgG | HUABIO | HA1006 | 1:50000 |
| HRP Conjugated Goat anti-rabbit IgG | HUABIO | HA1001 | 1:20000 |
| Anti-F4/80 | HUABIO | HA721520 | 1:500 |
| Anti-CD14 | HUABIO | ET1610-85 | 1 :500 |
